# Multidimensional Characterization of Parkinson’s Disease Subtypes Through Motor Neuron Excitability and Peripheral Immune Dynamics: Insights from F-Wave Modulation Metrics

**DOI:** 10.3390/diagnostics16010027

**Published:** 2025-12-22

**Authors:** Esra Demir Unal, Yiğit Emre Dagdelen

**Affiliations:** 1 Department of Neurology, Medical Faculty, Ankara Yıldırım Beyazıt University, Ankara 06800, Turkey; 2 Neurology Clinic, Ankara Bilkent City Hospital, Ankara 06800, Turkey

**Keywords:** proximal motor conduction, F-wave analysis, hematoinflammatory indices, mean platelet volume, motor subtypes, nerve conduction studies, neurophysiology, Parkinson’s disease, platelet–eosinophil index, systemic inflammatory response index

## Abstract

**Background/Objective:** Central pathophysiological heterogeneity among Parkinson’s disease (PD) motor subtypes has been increasingly recognized, yet subtype-specific peripheral disturbances are limited. We aimed to characterize demographic, biochemical, and neurophysiological differences among PD motor subtypes, evaluate hematoinflammatory effects on peripheral and proximal motor conduction, and identify prognostic phenotypic biomarkers. **Methods:** A total of 110 participants (60 idiopathic PD patients (30 akinetic-rigid (AR), 30 tremor-predominant (TD), and 50 age- and sex-matched healthy controls (HCs)) were enrolled. Demographic data, nerve conduction studies (NCS) including detailed F-wave analysis, and hematoinflammatory markers were collected. Kruskal–Wallis, linear mixed models, multivariable regression, and ROC analyses were applied. **Results:** Hematoinflammatory indices were elevated in both subtypes compared with HCs, with more pronounced changes in AR (mean platelet volume (MPV) H = 4.367, *p* = 0.003; systemic inflammatory response index (SIRI) H = 3.929, *p* = 0.004). AR showed severe upper-limb–predominant motor involvement (median motor onset latency H = 55.30, *p* < 0.001; amplitude H = 50.52, *p* = 0.04; conduction velocity H = 49.15, *p* < 0.001), whereas TD showed milder, lower-limb–predominant changes (tibial motor onset latency H = 19.89, *p* < 0.001; amplitude H = 51.50, *p* = 0.02; velocity H = 15.39, *p* < 0.001). AR also demonstrated prolonged minimal (Fmin)/mean (Fmean) ulnar F-wave latencies versus TD (respectively, H = 10.51, *p* = 0.001; H = 8.79, *p* = 0.003), with both showing increased tibial Fmean/Fmax latencies. Platelet–eosinophil indices independently predicted ulnar F-latencies (B = 0.104–0.105; *p* = 0.001; model R^2^ = 0.21–0.39). Select F-wave metrics yielded ROC AUCs ≈ 0.65–0.92 (ulnar Fmin AUC ≈ 0.92 vs. HCs); AR achieved sensitivity/specificity ≈ 70–74%. **Conclusions:** The AR subtype showed increased hematoinflammatory changes, specifically in MPV and SIRI, as well as a tendency toward more pronounced proximal motor and peripheral nerve conduction impairment compared with TD. Platelet–eosinophil indices and F-wave metrics may represent potential candidate markers for diagnostic or stratification purposes in PD subtyping and could possibly aid in prognostic estimation.

## 1. Introduction

Parkinson’s disease (PD) is the second most common neurodegenerative disorder, characterized by the progressive loss of dopaminergic neurons in the substantia nigra pars compacta [1]. The core motor symptoms are tremor, bradykinesia, rigidity, and postural instability; however, the disease exhibits considerable heterogeneity in terms of clinical presentation [2]. Tremor-dominant (TD) and akinetic-rigid (AR) subtypes, mainly based on motor dominance, are the most widely accepted classifications of PD in clinical practice [3]. These two phenotypes differ markedly not only in terms of motor symptom profile but also in clinical progression, cognitive course, and underlying pathophysiological processes. The TD phenotype is generally characterized by slower motor progression and better prognosis, while AR is associated with early postural instability, a tendency to fall, and rapid motor deterioration. Recent studies have shown that this phenotypic distinction is reflected not only in clinical observations but also in neurophysiological, biochemical, and systemic inflammatory profiles [2,3].

There is increasing evidence that peripheral neuropathy is more common in PD than in the general population: systematic estimates suggest that large-fiber neuropathy affects around ~16% of PD patients and small-fiber neuropathy up to ~57% [4]. For example, a recent cohort study of 99 PD patients found PN in 40.4%, with a predominance of small-fiber involvement (70%), and notably faster gait and balance impairment in those with neuropathy [5]. Despite this, it remains unclear whether the prevalence, severity, or subtype of PN differ by motor phenotype. The F wave is an important electrophysiological component that provides indirect information about the excitability of spinal motor neurons and spinal motor neuron pool excitability [5], but data on the pattern of involvement in PD are limited. Additionally, there is insufficient evidence to suggest that changes in F-wave parameters, such as minimum-maximum latency, persistence, and chronodispersion, reflect differences in proximal motor control mechanisms among motor subtypes of PD.

Recent biomarker studies have proposed hypotheses that peripheral inflammation and hematological parameters may differ across PD motor subtypes. For example, various hemo-inflammatory and systemic immune-inflammatory indices are higher in AR phenotypes and relatively lower in the tremor-dominant TD subtype [6,7,8]. Additionally, it has been reported that metabolic indicators, such as serum glucose and HbA1c levels, also differ between groups and may influence the rate of dopaminergic degeneration, leading to varying degrees of clinical heterogeneity across motor phenotypes [9]. However, comparative analyses integrating these hematologic–inflammatory and vasculopathic indices with electrophysiological parameters across PD motor subtypes remain insufficient, and the pathophysiological basis of these subtype-specific differences has yet to be systematically elucidated.

This study aimed to demonstrate the biochemical and electrophysiological differences both among the PD motor subtypes and between each subtype and HCs. The primary hypothesis is that the AR phenotype is characterized by more extensive neurological and systemic involvement compared to the TD. Based on this hypothesis, (1) abnormalities in systemic inflammatory markers and indices reflecting platelet activation or metabolic dysfunction; (2) peripheral neuropathic changes identified through motor and sensory nerve conduction studies; and (3) altered proximal motor conduction dynamics in F-wave analyses indicative of spinal motor neuron pool excitability shifts were expected to be more prominent in the AR group. Furthermore, both PD subtypes were hypothesized to differ significantly from HCs across these parameters. Secondary objectives include evaluating dopaminergic response and motor fluctuation profiles between PD subtypes and exploring the distribution of demographic and clinical comorbidities, particularly those related to vascular disease.

## 2. Materials and Methods

This cross-sectional prospective case–control study was conducted at a tertiary movement disorders center. Informed consent was obtained from all subjects involved in the study, and the study protocol was approved by the Institutional Ethics Committee of ANKARA CITY HOSPITAL (TABED 1/861/2025, 15 January 2025). Consecutive participants were recruited at the Ankara City Hospital Movement Disorders Clinic between January 2025 and April 2025. All clinical, laboratory and electrophysiological assessments were performed within this interval. To minimize selection bias and ensure the external validity of the findings, a consecutive sampling strategy was employed. All patients presenting to the movement disorders outpatient clinic during the study period were screened for eligibility. Eligible candidates who provided informed consent were enrolled sequentially, preventing the preferential selection of patients with specific symptom severities. A total of 60 patients with idiopathic PD, diagnosed according to the Queen Square Brain Bank criteria, and 50 age- and sex-matched healthy controls without neurological or systemic disorders were included. The PD group was clinically staged using the Movement Disorder Society–Unified Parkinson’s Disease Rating Scale, Part III (MDS-UPDRS III), and the Modified Hoehn and Yahr (mH&Y) scale, both administered during the “on-medication” state. Demographic and clinical characteristics, such as age, sex, height, weight, body mass index, age at onset, disease duration, MDS-UPDRS III score, mH&Y stage, and daily antiparkinsonian medication, were recorded. Data verification was independently performed by two investigators, and discrepancies were resolved by a third reviewer. To minimize heterogeneity related to recent treatment changes, eligibility required a stable dopaminergic regimen defined as no change in dopaminergic agent or dose for at least four weeks prior to the study visit. Participants were instructed to take their regular medications as prescribed on the day of testing; medications were not withheld for electrophysiological assessment. Drug-naïve patients were excluded from the study. For analytic control, levodopa equivalent daily dose (LEDD) was calculated for each participant using standard conversion tables and was included as a covariate in adjusted analyses, including the prespecified linear mixed-effects models. This approach ensured that electrophysiological measurements reflect participants’ typical clinical on-medication state while allowing statistical adjustment for differences in dopaminergic exposure.

*Inclusion criteria* were as follows: age between 18 and 80 years, diagnosis of idiopathic PD, mH&Y ≤ 2, MDS-UPDRS III ≤ 20, and the availability of complete laboratory and EMG/nerve conduction data. The mH&Y and MDS-UPDRS-III criteria refer to the initial clinical screening performed to determine eligibility. Motor ratings reported in the manuscript were recorded at the time of the electrophysiological study during the participant’s usual on-medication state; therefore, in a minority of cases, scores at the study visit were higher than the initial screening values due to interval progression or because screening ratings were performed at a separate clinical visit. We have made the screening versus visit timepoints explicit to avoid ambiguity. Motor subtypes were classified as TD or AR based on previously validated ratios [10]. We calculated a tremor-related component score as the mean of MDS-UPDRS items 2.10, 3.15, 3.16, 3.17 and 3.18 (patient-reported tremor and clinician-rated postural/kinetic/rest tremor items) and a gait/PIGD (postural instability/gait difficulty) score as the mean of items 2.12, 2.13, 3.10, 3.11 and 3.12 (walking and balance, freezing, gait and postural stability). The subtype ratio was computed as:

TD/PIGD ratio = (tremor-related components score)/(gait/PIGD score).

Predefined cut-offs were applied as in the original validation: ratio ≥ 1.15 → Tremor-Dominant (TD); ratio ≤ 0.90 → PIGD/AR (classified here as AR); ratio 0.90–1.15 → indeterminate/mixed. We prospectively included only participants who met the TD or AR cut-offs; participants with indeterminate ratios were not included in subtype-specific analyses. This procedure produced the two PD subgroups analyzed in this report. The method and cut-offs follow Stebbins et al. [10] and subsequent MDS-UPDRS mappings. Healthy controls underwent the same baseline clinical interview and neurological examination as PD subjects. Screening included review of past medical history, current medications, focused neurological examination to exclude parkinsonism or other focal neurological deficits, and basic laboratory screening, including complete blood count, fasting glucose, HbA1c, liver and renal function tests, etc.

*Exclusion criteria* included secondary or atypical parkinsonism, prior deep-brain stimulation, intestinal levodopa–carbidopa gel infusion, or apomorphine therapy. Subjects with significant psychiatric disorders, cardiovascular disease, or abnormal laboratory parameters were excluded. MRI or genetic findings suggestive of atypical parkinsonism or Parkinson-plus syndromes were also excluded. Participants with a prior diagnosis of neuropathy or with symptomatic clinical neuropathy at screening (history, documented prior NCS, or clinical signs such as distal sensory loss and distal weakness consistent with neuropathy) were excluded. Electrophysiological recordings performed for the study were used for objective characterization only and were not applied retrospectively to exclude participants. Thus, subclinical NCS abnormalities identified during study testing were retained in the dataset and analyzed, because one of the study aims was to quantify subclinical peripheral nerve involvement across PD subtypes. Controls with a history of neurologic disease, active systemic inflammatory disease, uncontrolled metabolic or cardiovascular disease, abnormal relevant laboratory results, or abnormal neurological examination were excluded.

Laboratory measurements were obtained from fasting blood samples; complete blood count, fasting glucose, and HbA1c analyses were performed at the institution’s accredited laboratory. Inflammatory indices were calculated under the literature as follows: NMR (Neutrophil/Monocyte Ratio), EMR (Eosinophil/Monocyte Ratio), BMR (Basophil/Monocyte Ratio), ERR (Eosinophil/RBC Ratio), NLR (Neutrophil/Lymphocyte Ratio), SII = ((Neutrophil × Platelet)/Lymphocyte), SIRI = ((Neutrophil × Monocyte)/Lymphocyte), AISI = ((Neutrophil × Monocyte × Platelet)/Lymphocyte). All electrophysiological recordings were conducted by the senior investigator (E.D.U.). Data acquisition and verification were independently cross-checked by a blinded secondary examiner (Y.E.D.) to ensure procedural reliability.

All nerve conduction studies (NCSs) and F-wave recordings were performed on a Natus Keypoint^®^ G4 workstation following our laboratory’s standard protocol by the senior investigator (E.D.U.) and independently cross-checked by a blinded examiner (Y.E.D.), and external reviewer. Surface Ag/AgCl electrodes were used for all recordings. Participants were seated comfortably during testing. Electrode placement followed standard anatomical landmarks: median motor—active electrode over the abductor pollicis brevis (APB) and reference over the tendon; ulnar motor—active over the abductor digiti minimi (ADM); tibial motor—active over the abductor hallucis (AH); peroneal (deep fibular) motor—active over the extensor digitorum brevis (EDB). Stimulating sites were the wrist and elbow for median/ulnar nerves, the ankle (medial malleolus/posterior tibial) and popliteal fossa for the tibial nerve, and the ankle and fibular head for the peroneal nerve. Distances between distal and proximal stimulation sites were measured individually along the skin using a flexible measuring tape to the nearest 0.1 cm and used for conduction velocity calculations.

*Stimulation frequency and intensity*: Routine motor and sensory NCS used single-pulse stimulation at 0.2–1.0 Hz depending on nerve and test type (this value had lower frequencies for sensory studies). F-wave acquisition used at least 20 supramaximal stimuli delivered at approximately 0.5–1.0 Hz; stimulus intensity for F-wave testing was set to 20–25% above the M-response plateau.

*Limb temperature calibration and control:* Skin temperature was measured with a calibrated surface probe at the recording site immediately prior to testing. We required skin temperature ≥32.0 °C for upper limbs and ≥31.0 °C for lower limbs; when temperature was below thresholds the limb was warmed using a heating pad or infrared lamp and re-measured until the target temperature was achieved. Pre- and post-recording temperatures were logged for each limb. These temperature thresholds and procedures were consistently applied across participants.

*Amplifier/filter and acquisition settings:* Recorded signals used a bandpass filter of 2 Hz–10 kHz and a sweep of 5 ms/div for motor studies; sampling and display settings were standardized for all subjects to permit direct comparison.

*Artifact detection and repeat rules:* All traces were visually inspected in real time. Traces were repeated if any of the following were observed: (a) stimulus artifact obscuring the onset of the M-response or F-wave; (b) electrical mains interference or baseline noise that exceeded ~5% of the peak-to-peak M-response amplitude; (c) M-response amplitude variability >10% between successive stimuli suggesting unstable electrode contact; or (d) movement or electrode displacement. If an individual nerve required more than three repeated attempts and a stable trace could not be obtained, the participant was excluded from the study.

*F-wave protocol*: At least 20 stimuli were applied to each motor nerve evaluated using supramaximal stimulation; stimulation intensity was selected to be 20–25% higher than the plateau point of the M-response. Recorded F-wave parameters were as follows: minimum (Fmin), mean (Fmean), and maximum (Fmax) F-latency (ms); chronodispersion (Fmax − Fmin, ms); persistence (%) = (number of F-responses/total number of stimuli) × 100; and mean F-wave duration (ms). The linear relationship between F-latency and height and age was taken into account for the evaluation.

Sample size determination was performed using G*Power 3.1.9.7 for the primary outcome, based on an expected between-group effect size of d = 1.247 (two-tailed, α = 0.05, power = 0.95) with a large effect size (Cohen’s d ≈ 0.50). The target effect size was derived from previously published electroneurophysiological studies in PD, resulting in a required sample size of 18 participants per group (total *n* = 36). Matrix/data analysis scripts (SPSS 29.0.2) were archived and retained for study reproducibility.

### Statistical Methods

All statistical analyses were performed using SPSS (version 29.0.2). Normality of continuous variables was assessed using the Shapiro–Wilk test; variables conforming to normality are reported as mean ± SD, and those not conforming to normality are reported as median (IQR). Homogeneity of variance was tested using the Levene test. Variables not conforming to normality were subjected to log or Box–Cox transformations. If normality was not achieved after transformation, nonparametric tests were applied. Between-group comparisons, if parametric assumptions were met, the independent samples *t*-test or ANOVA was used for univariate analyses; if assumptions were not met, the Mann–Whitney U or Kruskal–Wallis tests were used. The χ^2^ test or Fisher’s exact test was used for categorical variables. Appropriate adjustments were reported in post hoc analyses after pairwise or multiple comparisons. Because dependencies exist in the data due to bilateral EMG measurements and nerve-based repetitions, linear mixed models were used to model repeated measurements. A random intercept was added to the models and fixed effects included demographic data. F-wave parameters and clinical scores correlations, as well as inflammatory and metabolic indices, were calculated via Pearson or Spearman correlation coefficients. The contribution of independent effects in multivariate models was assessed using linear regression. For the multivariable regression models we report model fit (R^2^ range) and performed standard diagnostic checks: normality (Q–Q plots, Shapiro–Wilk), homoscedasticity (Breusch–Pagan test and residual vs. fitted plots), and influence diagnostics (Cook’s D and leverage). Variance inflation factors (VIF) are provided in the regression tables to document multicollinearity checks. The linear relationships of F-latencies with age (years), sex (binary), height (cm), disease duration (years) and LEDD (mg)were taken into account in the analyses. Full regression coefficients, standard errors, t-statistics, *p*-values and VIFs are presented in the Results tables.

The diagnostic performance of F-wave parameters was assessed using ROC analyses, and these analyses were conducted exclusively for F-wave parameters that demonstrated statistically significant intergroup differences in preliminary comparisons. Parameters without significant differences were excluded from the ROC modeling to avoid redundancy and ensure an analytical focus on clinically meaningful predictors. To mitigate potential selection bias, the inclusion criteria were determined based on prior variance analyses, and the resulting AUC and Youden index values were interpreted independently to evaluate diagnostic accuracy. Bonferroni correction was applied when necessary in multiple comparisons. The two-way significance level was accepted at *p* < 0.05.

The primary endpoint was predefined as the ulnar Fmin measured at the study visit. Ulnar Fmin was selected a priori because minimum F-latencies are sensitive indicators of proximal motor conduction delay and spinal motor neuron pool excitability, and our primary hypothesis anticipated between-phenotype differences. The prespecified primary inferential analysis was a linear mixed-effects model (LMM) to account for repeated/bilateral nerve measurements within subjects. The LMM specification was: dependent variable = ulnar Fmin (continuous); fixed effects = group (AR, TD, HC), side (left/right), age (years), sex (male/female), height (cm), disease duration (years) and LEDD; random effects = subject intercept to account for within-subject correlation; covariance structure = unstructured (or a parsimonious alternative if required for convergence); estimation = restricted maximum likelihood (REML). Pairwise contrasts for the prespecified primary comparison were estimated from the LMM as least-squares means and reported with 95% confidence intervals. Unadjusted Kruskal–Wallis/ANOVA results are retained in the tables for descriptive transparency, but inferential conclusions for the primary endpoint follow the prespecified LMM. Model fit and assumptions were assessed using standard residual diagnostics.

## 3. Results

### 3.1. Demographic and Clinical Parameters

Demographic characteristics are summarized in Appendix A.1. The cohorts exhibited homogeneity in baseline demographic variables. The AR cohort showed a statistically significantly higher concentration of comorbidities compared to both the TD and HC groups, particularly depression (χ^2^ = 30.11, *p* < 0.001) and psychosis (χ^2^ = 10.65, *p* = 0.005). In terms of cardiovascular comorbidities, the AR group had the highest prevalence of hypertension (χ^2^ = 13.38, *p* = 0.001). Furthermore, valvular heart disease (χ^2^ = 13.96, *p* = 0.001), which was not observed in the control group, stands out as a significant clinical burden in this group. Cardiac arrhythmias (χ^2^ = 8.63, *p* = 0.013) were detected statistically significantly more frequently in the TD group compared to both the AR and especially the control group. Similarly, chronic lung disease (χ^2^ = 7.40, *p* = 0.025), thyroid dysfunction (χ^2^ = 11.91, *p* = 0.003), and the use of lipid-lowering medications (χ^2^ = 17.39, *p* < 0.001) reached a statistically significant level in the TD group. In Appendix A.2, the comparative analysis of the clinical and treatment profiles of the motor phenotypes is presented. Both groups exhibit no significant differences in baseline demographic variables and core dopaminergic treatment exposure. The AR subtype demonstrates a significantly higher prevalence and severity in daily Off-time (U = 217, *p* < 0.001), functional impact of motor fluctuations (U = 216, *p* < 0.001), and complexity of these fluctuations (U = 232, *p* = 0.001) when contrasted with the TD cohort. Both mH&Y staging (U = 242, *p* = 0.001) and UPDRS-III motor scores (U = 267, *p* = 0.007) indicate markedly more severe motor impairment in the AR group. The prespecified primary analysis, including LLM model, REML estimation (dependent variable = ulnar Fmin; fixed effects = group, side, age, sex, height, disease duration and LEDD; random intercept = subject) produced a directionally consistent pattern (AR > TD > HC), but the AR versus TD contrast was attenuated and did not reach statistical significance after covariate adjustment.

### 3.2. Peripheral Immune and Hematologic Dynamics Across Groups

In comparative analysis of hematological and systemic immune-inflammatory markers, statistically significant overall group effect was observed, particularly in two composite parameters that reflect platelet activation and the systemic inflammatory response (Table 1).

#### 3.2.1. AR Phenotype Assessment

**Figure 1:**
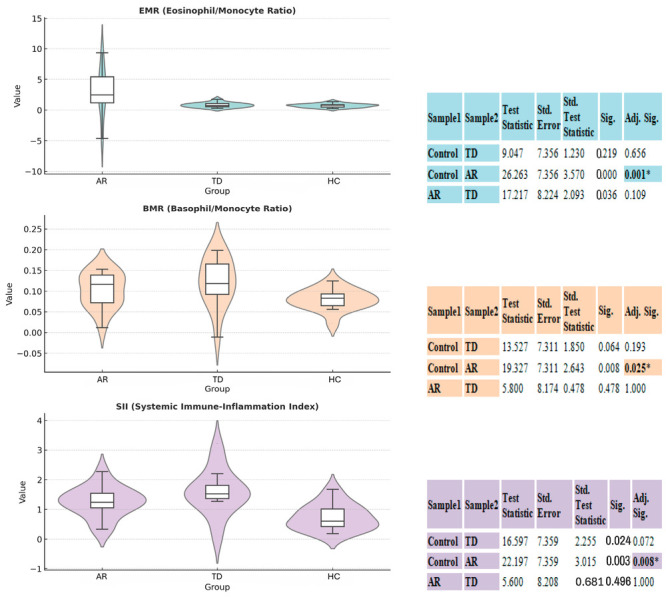
Comparison of inflammatory and hematological indices between akinetic-rigid (AR), tremor-dominant (TD), and control (HC) groups. Violin–box plots illustrate the distribution and density of EMR, BMR, and SIRI across the three groups. Scatter overlays indicate individual subject variability. On the right the boxplot, a table summarizes the results of Dunn’s Post Hoc Test with Bonferroni Correction. The overall Kruskal–Wallis H test indicated a significant difference between the groups (* *p* < 0.05), as noted. The post hoc test further specifies pairwise comparisons, showing significant adjusted *p*-values (Adj. Sig.).

The AR group exhibited a statistically significant immunological shift compared to the HC group with a SIRI value. The Kruskal–Wallis test showed that this difference reached statistical significance (*p* = 0.04). Also in Dunn’s Post Hoc analyses (Figure 1) the comparison between the HCs and AR was significant for the SIRI. Similarly, the other two inflammatory ratios shown, the EMR and BMR, also showed a statistically significant difference only between the HC group and the AR group. The MPV value is part of the overall variation and is numerically higher than in the HC group. The significance levels of the data are shown accordingly. This pattern, suggests that the AR subtype is the primary driver of subclinical immunoreactivity in the platelet and specialized leukocyte axis, rather than general inflammatory activation, demonstrating a consistent and robust differentiation compared to the HC group within the examined inflammatory ranges.

#### 3.2.2. TD Phenotype Assessment

There was overall Kruskal–Wallis significance in the SIRI and MPV in the TD group (SIRI *p* = 0.04, MPV *p* = 0.03). Post hoc analyses showed that although the TD group had numerically higher SIRI, EMR, and BMR values than HC and similar values to AR, none of these differences reached statistical significance (all Adj. Sig. < 0.05). These findings confirm that the TD phenotype also carries a specialized perturbation in systemic inflammation and platelet activation, but unlike AR, it does not reliably differentiate statistically from the HC group on these specific inflammatory ratios.

### 3.3. Quantitative Electrophysiological Characteristics of Groups

Inferential analysis utilizing the Kruskal–Wallis test on motor and sensory nerve conduction parameters across motor subtypes and HC cohorts confirms the presence of a neuropathic burden in PD patients (Table 2).

#### 3.3.1. Electrophysiological Characteristics of the AR Phenotype

The AR subtype exhibits the electrophysiological signature associated with the maximal temporal delay in upper extremity motor conduction. The prolongation in median motor nerve onset latency is numerically the highest among all groups, demonstrating a marked contrast to the HC group. The median motor NCV value represents the most severe conduction deceleration, being approximately half the speed of the HC cohort. In lower extremity motor pathways, peroneal motor NCV and tibial motor amplitude demonstrate lower quantifiable performance metrics compared to the TD group. These findings suggest that the AR group carries a quantifiable load of both demyelination and axonal loss components that is more pronounced than in the TD group. The significance levels of the data are shown in Table 2.

#### 3.3.2. Electrophysiological Characteristics of TD Phenotype

Significant deceleration is present in the median motor nerve NCV compared to HC. However, the lower extremity analysis reveals a maximal temporal delay in the tibial motor nerve onset latency, which is the longest across all cohorts, exceeding both AR and HC. This supports the hypothesis of a specific and disproportionate temporal disparity in the distal lower extremity motor pathways for the TD subtype. Amplitude data appear relatively better preserved in TD; tibial motor amplitude and peroneal motor amplitude are numerically higher than those observed in the AR group (for tibial nerve). Furthermore, the sural NCV value represents the slowest conduction velocity in the sensory system across all cohorts. This electrophysiological pattern suggests that while TD exhibits a generalized dysfunction, its quantitative impairment in overall axonal integrity may be less severe than that of AR, despite having specific segmental vulnerabilities. The significance levels of the data are shown in Table 2.

### 3.4. Quantitative Analysis of F-Wave Parameters

In the analysis of F-wave parameters, the AR group demonstrated quantitatively more pronounced proximal motor conduction and spinal motor neuron pool excitability pathway dysfunction (Table 3). The fact that the overall F-wave chronodispersion value remained invariant across all groups suggests that motor neuron pool recruitment efficiency was preserved in the TD and AR groups.

#### 3.4.1. Modeling of F-Wave Parameters in AR Phenotype

**Figure 2:**
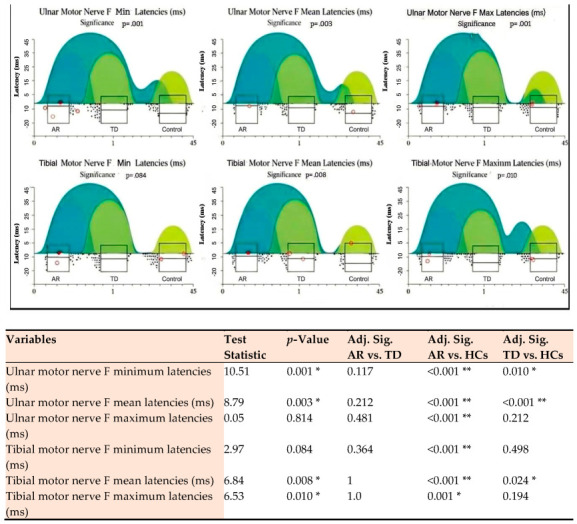
Post hoc analysis of ulnar and tibial motor nerve F-wave latencies across three groups. Significance is indicated as *p* < 0.05 (*) and *p* < 0.01 (**). AR: Akinetic-Rigid; TD: Tremor-Dominant; HC: Healthy Control. Adj. Sig.: Adjusted Significance). Each subplot shows the distribution of data points (scatter plot), density estimation (cloud/rain), and boxplots (median, interquartile range, outliers). *p*-values indicate the overall significance from a non-parametric test Kruskal–Wallis H test comparing the three groups. Color Legend: Dark Turquoise/Blue-Green: Represents the AR group; Medium Green: Represents the TD group; Light Green/Yellowish: Represents the Control group.

The AR phenotype exhibited a statistically significant prolongation of minimum latencies (H = 10.51, *p* = 0.001) and mean latencies (H = 8.79, *p* = 0.003) of the ulnar motor nerve in the upper extremity compared to the HC group. Dunn’s Post Hoc analysis in Figure 2 demonstrates that this difference stems from the AR group, with the strongest significance: the significance value for the minimum latency of ulnar F is less than 0.001 for AR compared to HC, and the same strong difference is observed for the Fmean latency value. This prolongation pattern is numerically greater than the corresponding median values of the TD group, indicating a higher burden of proximal motor conduction and spinal motor neuron pool excitability pathway dysfunction in the motor nucleus pathways. In the upper extremity, AR exhibits numerically longer median values for ulnar F minimum and mean latencies, carrying a more pronounced burden of proximal motor pathway dysfunction. Tibial motor nerve F mean (H = 6.84, *p* = 0.008) and maximum latencies (H = 6.53, *p* = 0.010) in the lower extremity were also significantly delayed compared to HC.

#### 3.4.2. Modeling of F-Wave Parameters in the TD Phenotype

The TD phenotype also exhibits a significant central conduction delay compared to the HC group, as indicated by ulnar motor nerve F-wave minimum and mean latencies (Table 3). Post hoc analysis in Figure 2 shows that the TD group reached statistical significance for the ulnar F-wave minimum latency, while this difference was less than 0.001 for the mean latency. However, the TD group exhibited a quantitatively less pronounced prolongation pattern than the AR group in these upper extremity latencies. Tibial motor nerve F-wave mean maximum latencies were significantly delayed in the TD group compared to the HC group, with the tibial maximum latency being quantitatively longer than the AR group. In contrast, although the TD group showed a numerically higher delay in lower extremity proximal conduction, the Post Hoc analysis did not find a statistically significant difference in the max level between the TD group and the HC group. For tibial F mean latency, the separation of the TD group from HC is within the significance limit (Adj. Sig. = 0.024). Finally, the separation between the TD and AR groups for tibial motor nerve F mean and maximum latencies was not statistically significant.

### 3.5. Multiple Linear Regression Analysis of F-Wave Parameters for Clinical and Biochemical Predictors

**Figure 3:**
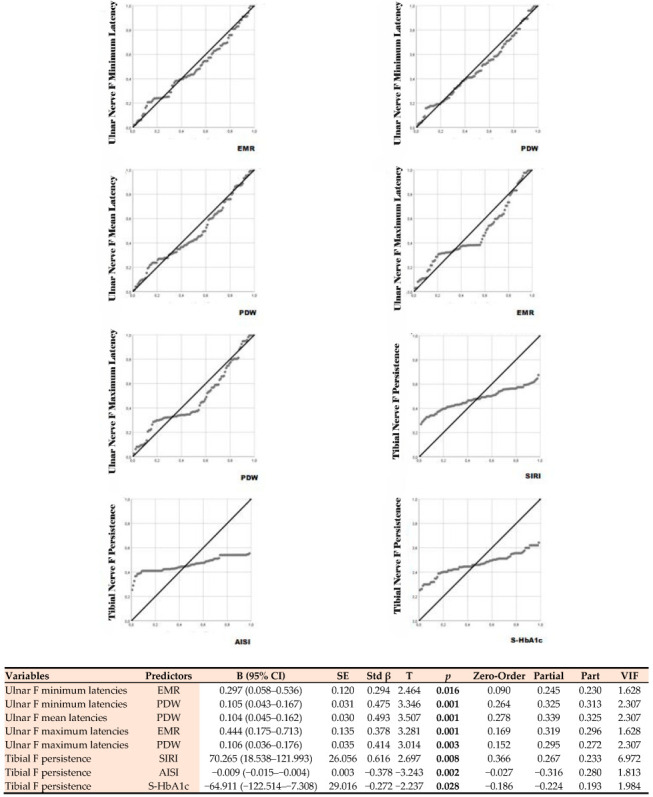
Linear regression analysis showing the relationship between F-wave parameters and biochemical predictors. EMR: Eosinophil-to-Monocyte Ratio; PDW: Platelet Distribution Width; SIRI: Systemic Inflammation Response Index, AISI: Aggregate Index of Systemic Inflammation, S-HbA1c: Glycated Hemoglobin A1c.

In a multiple linear regression analysis, the relationships between electrophysiological parameters and various clinical disability scores, as well as biochemical biomarkers, were evaluated (Figure 3). Models were constructed using the “enter” method, and analyses were repeated for both raw and transformed data, demonstrating the stability of the findings. Q–Q plots and the Shapiro–Wilk test were used to check the normality of variables, the Breusch–Pagan test and visual residual–fitted value plots were used to determine the homoscedasticity of residuals, tolerance and VIF (VIF < 2 in all models), and Cook’s D and leverage measures were used for influential observation/effect analyses. Furthermore, the Benjamini–Hochberg FDR correction was applied at the test level to mitigate multiple comparisons; the main findings remained significant after correction.

#### 3.5.1. Relationship with Clinical Disability Scores

Ulnar F minimum latency showed a positive significant relationship with the UPDRS Part III score (B = 0.0545; t = 2.615; *p* = 0.011). This finding indicates that a one-unit increase in motor score results in an average extension of the F minimum latency by 0.055 ms (95% confidence interval: 0.013–0.096; standardized β = 0.325). On the other hand, the analysis for F persistence revealed a negative and statistically significant relationship between the UPDRS Part III score and persistence (B = −0.380; t = −2.164; *p* = 0.034). This suggests a relationship between decreased excitability of the spinal motor neuron pool or decreased response sustainability of motor units. All other electrophysiological measurements and clinical variables examined did not show significant associations in the regression models.

#### 3.5.2. Relationship with Biochemical Predictors

The strongest independent predictor for ulnar F minimum latency was PDW, which had the highest standardized beta coefficient. Each unit increase in PDW corresponded to an average prolongation of the F minimum latency of 0.105 ms. The EMR variable also showed a significant positive relationship. Similarly, ulnar F mean latency was significantly predicted only by PDW. This indicates that the increase in PDW levels leads to a prolongation of the mean F latency. Ulnar F maximum latency was found to be positively correlated with both EMR. Ulnar F chronodispersion, which reflects the variation in peripheral nerve conduction, also showed a significant positive correlation with EMR and PDW. Tibial F minimum latency, representing the lower extremity, also exhibited significant correlations with EMR and PDW. The significance levels of the data are shown in Figure 3. Analysis results showed that the majority of F-wave measurements for both the ulnar and tibial nerves were significantly predicted by these biochemical variables. The explanatory power of the established models was found to be sufficient (R^2^ = 0.21–0.39; model *p* < 0.01), and no multicollinearity was observed (VIF < 2). The positive coefficients in all significant correlations indicate that increasing levels of biochemical markers are associated with prolonged F-wave latencies and increased chronodispersion.

### 3.6. Statistical Assessment of ROC and Heatmap Findings in F-Wave–Biomarker Associations

**Figure 4:**
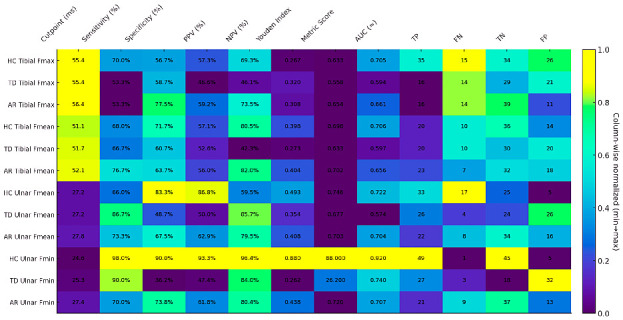
Heatmap illustrating the cut-off performance across study groups. Color intensity represents column-wise min–max normalization (0 = column minimum; 1 = column maximum), thereby reflecting the relative magnitude within each metric. Each cell displays the original, unnormalized value (percentages denoted by “%”, cut-points in milliseconds [ms], counts as integers, and indices/metrics with decimal precision). A higher Youden index (corresponding to stronger color intensity within its column) indicates superior discriminative performance for that variable/group. Abbreviations: AR, akinetic-rigid subgroup; TD, tremor-dominant subgroup; HC, healthy control; Fmin, F-wave minimum latency; Fmean, F-wave mean latency; Fmax, F-wave maximum latency; PPV, positive predictive value; NPV, negative predictive value; AUC, area under the ROC curve; TP, true positive; FN, false negative; TN, true negative; FP, false positive.

Heatmap analysis based on ROC analysis (Figure 4) demonstrates that F-wave parameters have the potential to discriminate among PD subtypes. The AUC values obtained were generally positioned in the “good” to “moderate” diagnostic accuracy range (0.65–0.92), suggesting that the parameters were significantly associated with disease phenotypes. When evaluated in conjunction with Youden indices, the determined cut-off points provide the optimal balance of sensitivity and specificity for each phenotype and can therefore be used as potential diagnostic thresholds in clinical practice.

*In the TD phenotype*, ulnar nerve F-wave parameters showed high sensitivity but relatively low specificity, suggesting that this subtype may be characterized by more widespread neurophysiological excitability, limiting the positive predictive power of the test. In contrast, tibial nerve parameters were more stable (AUC ≈ 0.59–0.60) but exhibited lower discriminatory power, suggesting that lower extremity motor units may be less significantly affected in the TD subtype.

*In the AR phenotype*, the ulnar nerve-derived Fmin and Fmean parameters exhibited a more balanced profile in terms of both sensitivity and specificity, with AUC values (≈0.70) found to be higher compared to the TD group. This suggests that the AR subtype yields a more homogeneous neurophysiological pattern in terms of F-wave parameters and provides more stable classification accuracy, as indicated by ROC analysis.

*In the HC group*, the ulnar motor nerve Fmin parameter exhibited a statistically significantly high discrimination power (AUC = 0.920, Youden = 0.880) and showed near-perfect performance in terms of both sensitivity and specificity.

## 4. Discussion

This study, to our knowledge, represents the first multimodal analysis integrating hematoinflammatory indices with motor and sensory nerve conduction studies and detailed F-wave parameters within the same cohort of PD motor subtypes. Our study’s findings revealed that the AR subtype exhibits marked proximal delay and conduction slowing in the upper extremities, accompanied by elevated systemic inflammation, particularly in MPV and SIRI, as well as a greater metabolic and vascular comorbidity burden. In contrast, the TD subtype demonstrates milder, predominantly lower-limb electrophysiological involvement with a slightly elevated hematoinflammatory profile. The platelet–eosinophil composite indices identification as independent predictors of ulnar F-latencies suggests a biologically meaningful link between systemic hematologic activity and proximal motor conduction integrity, which makes them of great importance for proximal motor conduction and spinal motor neuron pool excitability as potential biomarkers. In addition, this association has plausible pathophysiological relevance. One mechanistically coherent interpretation is that heightened platelet activation and low-grade systemic inflammation contribute to microvascular dysfunction, including endoneurial microcirculatory compromise, thereby rendering proximal peripheral nerve segments more susceptible to conduction slowing; alternatively or additionally, systemic inflammatory signaling may modulate spinal motor neuron excitability and reflex circuitry, which could be reflected in altered F-wave characteristics. These interpretations are offered as biologically plausible, hypothesis-generating links between systemic inflammatory/platelet measures and the electrophysiological phenotype; they do not imply proven causality and should be tested in longitudinal and mechanistic studies.

In our cohort, the AR group had a higher neuropsychiatric burden and greater cardiovascular load. In contrast, the TD group showed higher rates of arrhythmia, chronic lung disease, thyroid dysfunction, and lipid-lowering therapy (Appendix A.1). Overall comorbidity prevalence was significantly greater in AR than in TD and HC, supporting the view that non-motor symptoms in AR reflect more widespread limbic and cortical involvement of a more aggressive α-synucleinopathy [11,12]. The predominance of vascular and cardiac comorbidities in AR aligns with our hypothesis that this subtype is associated with accelerated vascular pathology and metabolic syndrome, underscoring the need for careful cardiovascular risk monitoring. The higher arrhythmia rate in TD offers a vital nuance: autonomic denervation in PD often begins at the cardiac level, and sympathetic denervation may be relatively pronounced in the TD phenotype [13,14], indicating subtype-specific autonomic patterns. Although dopaminergic exposure was similar across subtypes, AR patients had greater motor burden and complications and longer off-time, higher UPDRS-III and mH&Y scores, and more complex fluctuations constituting an “equal drug burden—different clinical outcome” pattern consistent with literature showing faster motor and cognitive progression, worse function, and more frequent motor complications in AR versus TD [15,16,17].

Table 1 data show limited but significant differences between all groups in terms of hematoinflammatory indices; SIRI (*p* = 0.04) and MPV (*p* = 0.03) were particularly significant. Post hoc analyses revealed significantly higher SIRI and MPV values in the AR group compared to the HC group, while similar trends, generally below the statistical thresholds, were observed in the TD group (Figure 1). The literature has reported that CBC-derived composite indices are associated with the progression of PD; specifically, SIRI can provide prognostic information on the inflammatory burden and neurodegenerative processes [18]. Similarly, platelet parameters such as MPV and PDW may be increased in neurodegenerative diseases and have been associated with microvascular/thromboinflammatory mechanisms [19]. Our findings suggest that the AR phenotype may represent a unique vasculoinflammatory biological signature accompanying dopaminergic neurodegeneration, a peripheral reflection of the platelet-inflammatory axis, and thus offer an innovative pathophysiological window for biomarker-based differentiation of PD subtypes. Emerging evidence links peripheral immune alterations and clinical phenotype in PD, suggesting that immune modulation may contribute to phenotype-specific trajectories; however, genotype–phenotype relationships remain incompletely defined and require dedicated genetic and mechanistic validation. Recent work reporting upregulation of PD-1 on peripheral T-cell subsets in PD supports the relevance of peripheral immune changes to disease biology, while contemporary reviews of non-ergot dopamine agonists highlight evolving therapeutic considerations that may differentially affect motor and non-motor features across phenotypes [20,21]. We therefore present our multimodal associations as hypothesis-generating and encourage further integrative studies that combine genetic, immunologic and longitudinal clinical data to clarify causal pathways.

Electrophysiological results indicate a distinct, widespread peripheral nervous system involvement in PD. A marked dissociation emerged between AR, TD, and HC groups in median motor nerve onset latency and conduction velocity (*p* < 0.001), suggesting distal demyelinating or mixed axonal–demyelinating processes. The more pronounced neuropathic alterations observed in the AR subtype further support the multisystem involvement hypothesis. A recent study by Kwon et al. [22] reported a high prevalence of large fiber neuropathy in PD. Our findings suggest that this neuropathy is more frequent and severe in the AR subtype, suggesting that this may be related to systemic inflammation, vascular risk factors, or disturbances in vitamin metabolism [23]. The literature has reported that decreased NCV and prolonged latency in the median and tibial nerves, associated with polyneuropathy in PD, are significant in PD patients. Some series have shown that lower extremity sensory deficits correlate with clinical functions, such as gait and balance, in PD [24]. Furthermore, similar NCS profiles are consistent with a pattern of demyelination and/or chronic axonal loss, and that levodopa-related metabolic factors, vascular comorbidity, and peripheral α-synuclein pathology may play a role in these changes [25,26]. The measurement sensitivity and statistical power of our findings indicate that these NCS differences are clinically meaningful; however, the heterogeneous nature of the distribution highlights the need for mechanistic disaggregation into neuropathy subtypes, metabolic screening, and longitudinal follow-up studies.

In this cohort, F-wave latencies reflecting proximal conduction showed significant delays in PD subtypes: ulnar F-minimum and F-mean latencies were significantly prolonged in both AR and TD groups compared to HC (*p* < 0.005). Similarly, significant delays were observed in tibial F-mean and F-maximum latencies in the lower extremities (*p* = 0.008 and *p* = 0.010, respectively). F-wave parameters reflect proximal motor conduction and spinal motor pool excitability; Fmin/Fmean latencies are generally interpreted as slowing of proximal conduction or a decrease in descending motor drive, while persistence and chronodispersion indicate the sustainability of recruitment of the motor pool [27]. In our study, the significant prolongation of ulnar Fmin and Fmean, along with the relative preservation of chronodispersion in the AR group, should be interpreted as indicating that the recruitment diversity of the motor neuron pool is relatively preserved, while proximal conduction velocity is reduced, and the descending inhibitory/excitatory balance is disrupted. This supports theories that rigidity and bradykinesia in AR may arise not only from basal ganglia dysfunction but also from degeneration of brainstem/spinal structures such as the reticular formation or spinal interneuronal networks [28,29]. Greater heterogeneity in motor-neuron synchronization observed in the AR subtype may reflect more widespread involvement of lower or upper-motor-neuron pathways, suggesting its potential as a biomarker. Post hoc analyses (Figure 2) revealed consistent, significant prolongation of ulnar F-min and F-mean latencies such that both AR and TD differed from HC, while AR vs. TD differences were non-significant. These findings imply that proximal conduction abnormalities are common across PD subtypes, with variation in severity rather than distinct subtype-specific signatures. Clinically, F-wave metrics therefore provide early electrophysiological discrimination of PD and contribute to subtype characterization. A comprehensive review by Beaulieu et al. revealed a similar heterogeneity/pattern in the prevalence and clinical impact of peripheral neuropathy in PD [30]. Regarding the lower extremity, the significant differences in tibial F-mean and F-max latencies, as indicated by post hoc analyses, were primarily due to the comparison between AR and control. Although similar tibial F-wave prolongations have been reported in various PD and related syndromes [31], the number of subtype-specific quantifications is limited. Our study provides an important quantitative contribution to fill this gap.

Our multiple regression models show that both platelet dynamics and EMR blood cell arrays make independent and significant contributions to the prediction of ulnar F-min and F-mean latencies (Figure 3). This pattern suggests that platelet activation/heterogeneity has a measurable effect on proximal motor conduction and that the eosinophil–monocyte axis may also play an immune-regulatory role. There are comprehensive reviews of similar platelet-neural relationships, as well as recent studies discussing the potential role of platelet biology in neurodegeneration [32]. In models for the lower extremities, the fact that SIRI was a strong positive predictor for tibial F-persistence suggests that inflammatory monocyte–neutrophil-mediated systemic load may affect the sustainability of motor unit responses; whereas, the negative association of AISI indicates a more complex picture, possibly showing multiple cellular interactions. The potential clinical use of SIRI and AISI has recently received increasing attention, supporting the hypothesis that they may be related to aspects of the non-motor and motor course in PD [33]. Furthermore, the negative association between tibial F-persistence and S-HbA1c suggests a complex relationship between metabolic control and motor unit responses; Because there is extensive literature suggesting that elevated HbA1c is typically associated with peripheral neuropathy [34], subanalyses disaggregating glycemic control and fine-tuning for the presence of diabetes are necessary for mechanistic dissociation of this finding. Our heatmap/ROC (Figure 4) shows F-wave metrics are strongly informative for PD phenotyping but insufficient alone. Ulnar F-min has high discrimination versus controls, indicating proximal conduction slowing, while ulnar F-min/F-mean yield balanced sensitivity–specificity in the AR subtype, suggesting a relatively homogeneous electrophysiological signature. By contrast, TD displays high sensitivity but low specificity, implying frequent but nonspecific F-wave positivity (more false positives). These findings align with prior work showing F-waves reflect proximal segment and spinal motor-pool changes. Recent reviews have provided evidence that platelet biology and CBC-derived inflammation indices are associated with neurodegeneration, significantly increasing the explanatory power of multimarker panels [35,36,37]. Furthermore, for ROC-based thresholds to be clinically applicable, retesting of these cutoff points in independent, multicenter, and longitudinal cohorts is essential; the literature clearly indicates that biomarker performance exhibits cohort-dependent variability and that external validation is mandatory [38].

### Limitations

This study offers novel insights into the peripheral biochemical and neurophysiological axis; however, several limitations should be acknowledged. The cross-sectional design precludes causal inference, and although the sample size is balanced across subtypes, it limits the generalizability of the findings. Validation in larger, prospective longitudinal cohorts is required to confirm the association between baseline platelet–eosinophil and electrophysiological profiles and objective progression rates. Additionally, the absence of neuroimaging or molecular inflammatory markers constrains mechanistic interpretation. Future studies integrating multimodal biomarkers and electrophysiological parameters are proposed.

## 5. Conclusions

Our study indicates that PD motor subtypes are biologically distinct: the AR phenotype exhibits a notably elevated burden, characterized by higher MPV and SIRI, greater metabolic and vascular comorbidity, and more pronounced proximal motor conduction abnormalities on F-wave analysis, along with peripheral NCS abnormalities. In contrast, the TD subtype demonstrates comparatively milder changes. Notably, platelet–eosinophil composite indices emerged as independent predictors of ulnar F-latencies in multivariable models, highlighting their added value beyond conventional hematoinflammatory markers. Mechanistically, this axis may capture the intersection of platelet activation, vascular risk, and eosinophil-mediated immune activity processes that can influence microvascular integrity and neuronal excitability, thereby providing a biologically plausible link to proximal motor conduction and spinal motor neuron pool excitability dysfunction. Through this, it may potentially represent an improvement in subtype discrimination, possibly offering candidate diagnostic or stratification markers that could help classify PD patients and prognosticate their clinical trajectories.

## Figures and Tables

**Table 1:** Group-wise descriptive statistics of hematologic and inflammatory biomarkers.

Variable	AR	TD	HC	Test Statistics (H)	*p*
NMR	12.6 (4.9–25.2)	12.1 (6.4–21.4)	10.7 (6.5–16.6)	0.004	0.94
EMR	0.4 (0–16.5)	0.3 (0–1)	0.2 (0.1–0.7)	0.953	0.32
BMR	0.1 (0–0.3)	0.1 (0–0.2)	0 (0–0.2)	3.038	0.08
ERR	0 (0–0.1)	0 (0–0.1)	0 (0–0.1)	0	1.00
NLR	2.5 (1.1–6.1)	2.2 (1.2–8.0)	1.8 (1.5–3.9)	0.802	0.37
PDW	50.4 (15.9–68.3)	45.7 (33–62.7)	50.4 (16.5–55.9)	1.985	0.15
MPV	8.3 (7.1–11.1)	8.2 (7.5–9.1)	7.9 (6.8–10.2)	4.367	0.03 *
SII	530 (184.9–1615)	459.7 (86.5–2810.7)	378.3 (295.8–2487.2)	0.715	0.39
SIRI	1.0 (0.2–1.6)	1.0 (0.4–6.3)	0.7 (0.3–1.9)	3.929	0.04 *
WBC	6.9 (3.6–10.2)	6.3 (3.9–12.8)	6.5 (6.0–7.5)	0.976	0.32
AISI	233 (8.2–870.7)	213.4 (71–23,302)	136 (92.3–1716.2)	0.634	0.42
S-Glucose	102.5 (75–115)	98.0 (80–108)	98.0 (85–107)	0.229	0.63
S-HbA1c	5.4 (4.46–6.50)	5.3 (4.40–5.80)	5.4 (4.8–5.7)	0.436	0.50

Data are presented as median (interquartile range). Between-group differences were analyzed using the Kruskal–Wallis H test. * *p* < 0.05 was considered statistically significant. AR: Akinetic-Rigid; TD: Tremor-Dominant; HC: Healthy Control; NMR: Neutrophil-to-Monocyte Ratio; EMR: Eosinophil-to-Monocyte Ratio; BMR: Basophil-to-Monocyte Ratio; ERR: Eosinophil-to-Red Blood Cell Ratio; NLR: Neutrophil-to-Lymphocyte Ratio; PDW: Platelet Distribution Width; MPV: Mean Platelet Volume; SII: Systemic Immune-Inflammation Index (Neutrophil × Platelet/Lymphocyte); SIRI: Systemic Inflammation Response Index (Neutrophil × Monocyte/Lymphocyte); AISI: Aggregate Index of Systemic Inflammation (Neutrophil × Monocyte × Platelet/Lymphocyte); WBC: White Blood Cell Count; S-Glucose: Serum Glucose; S-HbA1c: Glycated Hemoglobin A1c.

**Table 2:** Quantitative electrophysiological characteristics across groups.

Variables	AR	TD	HC	Test Statistics	*p*
Median motor nerve onset latency (ms)	11.1 (3.2–15.9)	10.2 (4.5–18.3)	3.3 (2.3–4.6)	55.30	<0.001 **
Median motor nerve amplitude (mV)	9.8 (7.8–11.8)	10.6 (8.6–12.6)	11.3 (4.1–18.8)	50.52	<0.04 *
Median Motor Nerve NCV (m/s)	28.4 (22.4–57.7)	27.6 (18.5–54.1)	57.5 (40.7–69.1)	49.15	<0.001 **
Ulnar motor nerve onset latency (ms)	2.5 (1.7–3.2)	2.3 (2.0–3.2)	2.4 (1.6–3.1)	0.03	0.846
Ulnar motor nerve amplitude (mV)	11.9 (6.3–16.7)	11.9 (7.6–19.3)	12.1 (3.6–19.2)	0.02	0.870
Ulnar motor nerve NCV (m/s)	54 (50.9–72.3)	56.8 (43.9–74.6)	59.0 (50.2–69.8)	2.28	0.131
Tibial motor nerve onset latency (ms)	5.7 (2.1–26.9)	6.2 (0.9–14.9)	4.1 (2.5–5.9)	19.89	<0.001 **
Tibial motor nerve amplitude (mV)	8.3 (3.7–12.9)	9.8 (4.8–14.8)	10.2 (5–8.1)	51.50	0.02 *
Tibial motor nerve NCV (m/s)	50.3 (37.3–60.9)	49.4 (32.3–61.7)	44.1 (34.8–57.4)	15.39	<0.001 **
Peroneal motor nerve onset latency (ms)	3.9 (0.4–9.4)	4.3 (1.93–8.8)	4.1 (2.6–5.6)	0.004	0.948
Peroneal motor nerve amplitude (mV)	4.4 (1.7–7.2)	5.5 (2.3–8.8)	6.15 (2.7–9.6)	54.21	0.44
Peroneal motor nerve NCV (m/s)	38.5 (20.0–57.1)	40.2 (25.2–55.5)	44.5 (38.8–57.1)	51.96	0.03 *
Median sensory nerve onset latency (ms)	9.6 (2.1–17.3)	13.3 (2.1–29.7)	2.4 (1.7–3.1)	51.18	0.127
Median sensory nerve amplitude (mV)	9.6 (5.5–13.8)	13.3 (8.5–18.1)	11.6 (10.5–12.8)	49.77	1.72
Median sensory nerve NCV (m/s)	31.9 (10.5–53.4)	32.9 (10.5–55.3)	49.3 (46.6–52.1)	51.08	0.178
Ulnar sensory nerve onset latency (ms)	2 (1.5–3.5)	1.9 (1.5–3.2)	1.7 (1.3–2.9)	1.04	0.30
Ulnar sensory nerve amplitude (mV)	9 (2.2–15.8)	10.5 (3.4–23.9)	12.2 (3.9–41.3)	1.05	0.30
Ulnar sensory nerve NCV (m/s)	53.6 (34.1–68.5)	50.6 (37.0–65.8)	53.7 (46.6–72.5)	3.68	0.05 *
Sural nerve onset latency (ms)	2.5 (1.6–3.3)	2.2 (1.8–6.6)	2.4 (1.6–3.3)	3.45	0.06
Sural nerve amplitude (mV)	11.6 (3.9–26.7)	14.6 (6.3–31.6)	11.5 (7.8–30.7)	1.19	0.27
Sural nerve NCV (m/s)	44.2 (35–57)	41.8 (15.0–54.1)	45.5 (15.0–60.6)	9.92	0.001 *

Data are presented as median (interquartile range). Between-group differences were analyzed using the Kruskal–Wallis H. Test Significance is indicated as *p* < 0.05 (*) and *p* < 0.01 (**). AR: Akinetic-Rigid; TD: Tremor-Dominant; HC: Healthy Control.

**Table 3:** Group comparison of F-wave electrophysiological measures.

Variables	AR	TD	HC	Test Statistics	*p*
Ulnar Motor Nerve F Minimum Latencies (ms)	28 (22.9–34.9)	27.1 (22.8–35.1)	25.2 (21.3–30.7)	10.51	0.001 **
Ulnar Motor Nerve F Mean Latencies (ms)	29.2 (23.6–35.3)	27.6 (24.1–36.4)	26.9 (22.6–32.3)	8.79	0.003 *
Ulnar Motor Nerve F Maximum Latencies (ms)	30.5 (25.3–38.7)	28.4 (25.5–39.7)	28.6 (23.7–34.6)	0.05	0.814
Ulnar Motor Nerve F Chronodispersion	2 (0.6–10.8)	1.95 (−3.11–1.0)	2.4 (0.4–7.8)	3.24	0.071
Ulnar Motor Nerve F Persistence	0.8 (0.1–1)	0.8 (0.6–1)	0.7 (0.5–1)	0.02	0.883
Tibial Motor Nerve F Minimum Latencies (ms)	51.8 (37.3–60.9)	49.4 (32.3–61.7)	47.2 (39.3–60.9)	2.97	0.084
Tibial Motor Nerve F Mean Latencies (ms)	53.3 (38.6–62.2)	52.9 (33.2–65.6)	49.4 (40.0–63.3)	6.84	0.008 *
Tibial Motor Nerve F Maximum Latencies (ms)	55.9 (39.9–68.3)	56.9 (35.4–77.0)	52.4 (40.6–66.0)	6.53	0.010 *
Tibial Motor Nerve F Chronodispersion	3.8 (1.1–24.7)	4.0 (0.5–16.9)	2.3 (0.8–13.6)	3.10	0.077
Tibial Motor Nerve F Persistence	0.9 (0.4–1)	0.8 (0.7–0.9)	0.8 (0.5–1)	0.14	0.703

Data are presented as median (interquartile range). Between-group differences were analyzed using the Kruskal–Wallis H. Test significance is indicated as *p* < 0.05 (*) and *p* < 0.01 (**). AR: Akinetic-Rigid; TD: Tremor-Dominant; HC: Healthy Control.

## Data Availability

The original contributions presented in this study are included in the article. Further inquiries can be directed to the corresponding author.

## Abbreviations

The following abbreviations are used in this manuscript:

PD	Parkinson’s disease
AR	Akinetic-Rigid
TD	Tremor-Dominant
HCs	Healthy Controls
NCS	Nerve Conduction Studies
CBC	Complete Blood Count
HbA1c	Glycated Hemoglobin A1c
MDS-UPDRS III	Movement Disorder Society–Unified Parkinson’s Disease Rating Scale Part III
mH&Y	Modified Hoehn and Yahr scale
EMG	Electromyography
NMR	Neutrophil/Monocyte Ratio
EMR	Eosinophil/Monocyte Ratio
BMR	Basophil/Monocyte Ratio
NLR	Neutrophil/Lymphocyte Ratio
ERR	Eosinophil/RBC Ratio
RBC	Red Blood Cell
MPV	Mean Platelet Volume
PDW	Platelet Distribution Width
SII	Systemic Immune-Inflammation Index
SIRI	Systemic Inflammation Response Index
AISI	Aggregate Index of Systemic Inflammation
WBC	White Blood Cell Count
S-Glucose	Serum Glucose
Fmin	F-wave minimum latency
Fmean	F-wave mean latency
Fmax	F-wave maximum latency
IQR	Interquartile Range
SD	Standard Deviation
VIF	Variance Inflation Factor
ROC	Receiver Operating Characteristic
AUC	Area Under the ROC Curve
PPV	Positive Predictive Value
NPV	Negative Predictive Value
TP	True Positive
FN	False Negative
TN	True Negative
FP	False Positive
FDR	False Discovery Rate

## Appendix A

### Appendix A.1

**Table A1:** Demographic Characteristics of the Study Group.

Variable	AR	TD	HC	Test Statistics	*p*-Value
Age, years (mean ± SD)	66.7 ± 1.37	66.9 ± 1.7	66.1 ± 0.9	H = 0.394	0.530
Female, *n* (%)	10 (33.3%)	12 (40.0%)	16 (32.0%)	0.558	0.757
Smoking history, *n* (%)	7 (23.3%)	10 (33.3%)	20 (40.0%)	2.34	0.311
Drinking history, *n* (%)	1 (3.3%)	0 (0.0%)	4 (8.0%)	2.90	0.234
Congestive heart failure, *n* (%)	3 (10.0%)	6 (20.0%)	8 (16.0%)	1.16	0.557
Cardiac arrhythmias, *n* (%)	4 (13.3%)	8 (26.7%)	2 (4.0%)	8.63	0.013
Valvular disease, *n* (%)	8 (26.7%)	6 (20.0%)	0 (0.0%)	13.96	0.001 *
Pulmonary circulation disorders, *n* (%)	2 (6.7%)	2 (6.7%)	7 (14.0%)	1.63	0.443
Peripheral vascular disorders, *n* (%)	9 (30.0%)	8 (26.7%)	9 (18.0%)	1.70	0.426
Hypertension, *n* (%)	21 (70.0%)	19 (63.3%)	16 (32.0%)	13.38	0.001 *
Chronic pulmonary disease, *n* (%)	1 (3.3%)	5 (16.7%)	1 (2.0%)	7.40	0.025
Liver disease, *n* (%)	1 (3.3%)	0 (0.0%)	6 (12.0%)	5.16	0.076
Thyroid dysfunction, *n* (%)	5 (16.7%)	7 (23.3%)	0 (0.0%)	11.91	0.003 *
Psychosis, *n* (%)	13 (43.3%)	3 (10.0%)	9 (18.0%)	10.65	0.005 *
Depression, *n* (%)	14 (46.7%)	4 (13.3%)	0 (0.0%)	30.11	<0.001 **
Use of lipid-lowering drugs, *n* (%)	3 (10.0%)	9 (30.0%)	0 (0.0%)	17.39	<0.001 **

Continuous variable was analyzed using the Kruskal–Wallis (H) test, while categorical variables were compared using the Chi-square (χ^2^) or Fisher’s Exact tests when appropriate. * *p* < 0.05 = statistically significant ** indicates *p* < 0.01.AR: Akinetic-Rigid; TD: Tremor-Dominant; HC: Healthy Control.

### Appendix A.2

**Table A2:** Clinical and Treatment Characteristics of PD Subtypes.

Variable	AR	TD	Test Statistics	*p*-Value
Disease duration, years	4.50 (2–8)	5.0 (2–10)	U = 440	0.888
Family history, *n* (%)	1 (3.3%)	3 (10%)	χ^2^ = 1.07	0.301
Dopamine dysregulation syndrome, *n* (%)	3 (10%)	3 (10%)	χ^2^ = 3.906	0.142
Levodopa-containing drug, score	2.30 (1–2.30)	1.20 (0.75–2.30)	U = 315	0.40
Dopamine agonist, score	1.0 (0–1)	1.0 (0–1)	U = 381	0.231
MAO-B inhibitor, *n* (%)	24 (80%)	25 (83%)	χ^2^ = 0.11	0.739
Amantadine, *n* (%)	10 (33%)	4 (13%)	χ^2^ = 3.35	0.067
Daily levodopa dose (mg)	544.5 (357.7–684)	519 (281.2–688.7)	U = 405	0.509
Daily levodopa-equivalent dose (mg)	300.00 (100–450)	300.00 (175–400)	U = 428	0.749
Off-time (h/day)	2.00 (0–6)	2.00 (0–2)	U = 217	< 0.01 **
Off-state dystonia (h/day)	0.00 (0–1)	0.00 (0–0)	U = 373	0.091
Time with dyskinesias (h/day)	0.00 (0–0)	0.00 (0–0)	U = 390	0.133
Functional impact of dyskinesias	0.00 (0–0)	0.00 (0–0)	U = 384	0.098
Functional impact of fluctuations	2.00 (0–3)	2.00 (0–1)	U = 216	< 0.01 **
Complexity of motor fluctuations	2.00 (0–2)	0.00 (0–1)	U = 232	< 0.01 *
Hoehn and Yahr stage	3.00 (2–3)	3.00 (1–2)	U = 242	< 0.01 *
UPDRS-III (motor score)	30.00 (8.7–44.2)	10.50 (8–23)	U = 267	< 0.01 **

U = Mann–Whitney U test (non-parametric); χ^2^ = Pearson chi-square test. Values are presented as median (Interquartile range) for continuous variables, and as counts and percentages for categorical variables * *p* < 0.05 = statistically significant ** indicates *p* < 0.01. AR: Akinetic-Rigi; TD: Tremor-Dominant.
